# Education, Occupation, and the Cognitive Performance Distribution of South Korean Older Adults

**DOI:** 10.1093/geroni/igab046.550

**Published:** 2021-12-17

**Authors:** Katherine Ford, Anja Leist

**Affiliations:** 1 University of Luxembourg, Esch-sur-Alzette, Luxembourg; 2 University of Luxembourg, Esch-sur-Alzette, Diekirch, Luxembourg

## Abstract

Earlier research suggests that educational attainment up to early adulthood are crucial for the development of cognitive reserve, while intellectually stimulating activities later in the life course are of limited impact. We sought to explore the effects of educational attainment and occupational factors (occupation type and currently having work) across the distribution of cognitive performance for adults aged 45-65 years in South Korea. We analysed scores from the Korean Mini Mental State Exam (MMSE) provided in the 2006 wave of the Korean Longitudinal Study of Aging. We used quantile regressions to both investigate relationships across the distribution and to reduce bias for measures of the central tendency as the MMSE is known for its ceiling effects. The quantile function at the lowest conditional decile of MMSE scores suggested that education level was the dominant significant factor for adult performance on the MMSE (lowest MMSE decile, primary education: β = 6.11 points, p < 0.001; secondary education β = 9.56 points, p < 0.001). All occupational factors were non-significant. Further factors with a significant association with the MMSE were hearing loss, the log-transformed household income, and age squared. With the conditional median function, occupational factors became significant in the middle of the distribution but remained much less important than education levels. In summary, educational levels were more important to explain variation in cognitive functioning than occupational factors, echoing studies with Western samples. We discuss the findings with regard to the historically gender unequal educational and occupational opportunities in Korea.

